# Liquid Metal Phagocytosis: Intermetallic Wetting Induced Particle Internalization

**DOI:** 10.1002/advs.201700024

**Published:** 2017-04-24

**Authors:** Jianbo Tang, Xi Zhao, Jing Li, Yuan Zhou, Jing Liu

**Affiliations:** ^1^ Department of Biomedical EngineeringSchool of MedicineTsinghua UniversityBeijing100084China; ^2^ Key Laboratory of CryogenicsTechnical Institute of Physics and ChemistryChinese Academy of SciencesBeijing100190China; ^3^ Key Laboratory of Photochemical Conversion and Optoelectronic MaterialsTechnical Institute of Physics and ChemistryChinese Academy of SciencesBeijing100190China

**Keywords:** interfacial physics, liquid metal, particles, phagocytosis, wetting and spreading

## Abstract

A biomimetic cellular‐eating phenomenon in gallium‐based liquid metal to realize particle internalization in full‐pH‐range solutions is reported. The effect, which is called liquid metal phagocytosis, represents a wet‐processing strategy to prepare various metallic liquid metal‐particle mixtures through introducing excitations such as an electrical polarization, a dissolving medium, or a sacrificial metal. A nonwetting‐to‐wetting transition resulting from surface transition and the reactive nature of the intermetallic wetting between the two metallic phases are found to be primarily responsible for such particle‐eating behavior. Theoretical study brings forward a physical picture to the problem, together with a generalized interpretation. The model developed here, which uses the macroscopic contact angle between the two metallic phases as a criterion to predict the particle internalization behavior, shows good consistency with experimental results.

From printer ink to mud avalanche, liquid‐particle mixtures represent a group of frequently encountered transitional‐state materials that differ largely from either liquid or solid.^1^, ^2^ The validity of dispersing particles into liquids to produce superior mixtures has been tested in systems such as nanofluids.^3^ This route gives rise to a process called particle internalization. Any child building sandcastles realizes it is effortless to mix sand with water. And perhaps it is because of this reason that the particle internalization process in conventional liquids is generally overlooked. For liquid metals (LMs), such problem becomes very important and also challenging due to the uniqueness of the base liquids rising from their surface tension, passivity, and many other aspects.^4^, ^5^, ^6^, ^7^ The combination of fluidity and metallic nature of LMs makes them appealing for applications where excellent heat conductivity, electrical conductivity, and flexibility are required.^8^, ^9^, ^10^ Up to now, the search for safe‐handling room‐temperature LMs only narrows down to gallium‐based LMs, a group of alloys consisting gallium and other metals like indium, tin, etc. Recently, self‐powered motor,^11^ jumper,^12^ oscillator,^13^ soft actuators,^14^, ^15^, ^16^ and 2D semiconductor skin^17^ demonstrated with this class of material shed light on their new possibilities toward more diverse applications. Here, we report a particle‐eating phenomenon of gallium‐based LMs, which mimics the biological phagocytosis process (also known as cellular‐eating, a basic cell behavior referring to the transportation of external particles into cells from across the membranes^18^, ^19^, ^20^, ^21^), and thus we call it LM‐Phagocytosis. Such phenomenon represents a wet‐processing strategy to achieve particle internalization. Therefore, the findings are expected to have both scientific and technological significance in preparing LM‐particle mixtures with enhanced properties or even new features.

In **Figure** **1**, we demonstrated that LM‐Phagocytosis with microparticles (MPs) and nanoparticles (NPs) made of copper could be realized in aqueous solutions covering a full‐pH range, i.e., from acidic solution (HCl solution) to neutral solution (NaCl solution) and to alkaline solution (NaOH solution). Droplets of the LM (eutectic gallium indium, eGaIn) were coated with MPs and NPs by rolling the droplets on the particle beds to fabricate LM marbles.^22^ When HCl solution was introduced, the particles first changed their tarnish appearance to pure‐copper red (more evident in the NPs case) and the nonspherical marbles transformed to highly spherical ones. Afterward, the LM marbles started engulfing the particles coating on the surface, accompanying with bubble generation.^12^ The phagocytosis (or equivalently, particle internalization) process came to an end (typically within 20 s) with the particles being internalized and the luster of bare LM being revealed (Figure 1a and Movies S1 and S2 of the Supporting Information). When NaCl solution or NaOH solution was used instead of the HCl solution, similar particle‐eating behavior was not observed (Figure S1, Supporting Information). But we later found that a cathode polarization voltage of 2.0 V or more (electrolysis of the solution became more intense at higher voltages) was effective to trigger LM‐Phagocytosis in the NaCl solution (Figure 1b and Movies S3 and S4 of the Supporting Information) and the NaOH solution (Figure S2a and Movies S5 and S6 of the Supporting Information). Given that a 2.0 V cathode voltage was an electrode potential that could be obtained with metals such as aluminum and magnesium in an alkaline solution, we proposed the third method of using a sacrificial metal to activate LM‐Phagocytosis in an alkaline environment. This method was verified when we placed the aluminum‐flake‐embedded LM marbles in the NaOH solution (Figure 1c and Movies S7 and S8 of the Supporting Information). By comparing the time spent to intake the particles, we noticed that the electrical polarization (the sacrificial metal served as an electrical polarization of its own electrode potential) exerted a much stronger impact on LM‐Phagocytosis than the acidic solution and it reduced the timescale from more than 10 s to only a few seconds. In addition, noticeable black product was observed in the NaCl solution after particle internalization but no such remainder was found in either HCl solution or NaOH solution. These evidences indicated that despite the similar results (particle internalization) brought by the three methods, there existed essential differences among different cases caused by the pH value of the solutions.

**Figure 1:**
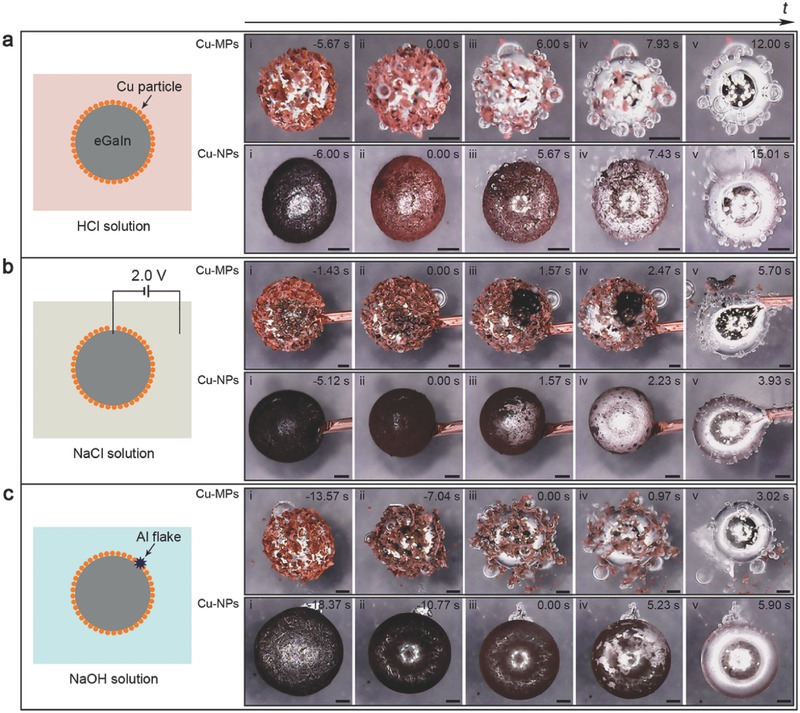
Methods to realize LM‐Phagocytosis in different solutions: a) acidic solution with no additional assistance; b) neutral solution assisted by an electrical polarization; c) alkaline solution assisted by an aluminum flake. In each figure, the schematic drawing shows the experimental arrangements and the cases concerned with Cu‐MPs, and Cu‐NPs in each solution are presented. The subfigures i–v in each row are time‐lapse images extracted from the movies with zero time indicating the beginning of phagocytosis. Scale bars: 200 µm.

So what is the science behind the observed LM‐Phagocytosis shared by all the cases? Bearing this question in mind, we at first considered the LM‐Phagocytosis to be a complete wetting behavior of the LM to the particles, and therefore we intended to investigate the relationship between wetting and particle internalization.^23^ Given the difficulties in directly measuring the contact angle of MPs and NPs, we switched to the problem of spreading an LM droplet on a substrate made of the same material as the particles. During investigating droplet on‐plate spreading, we found the LM wet copper substrate easily in HCl solution, which caused spontaneously spreading and neck formation. The stretching force resulting from the wetting was so strong that it further induced a neck‐breakdown and the droplet established a meniscus profile eventually (**Figure** **2**a, and Movie S9 of the Supporting Information). On the contrary, the LM formed large‐contact‐angle (over 160°) sessile droplets when the tests were conducted in both the NaCl solution and the NaOH solution. By introducing the same excitations that had been used to trigger LM‐Phagocytosis, i.e., an electrical polarization and a sacrificial metal, large (over 100°) and fast (within a few seconds) contact‐angle alterations were induced in the NaCl solution (Figure 2b, and Movie S10 of the Supporting Information) and the NaOH solution (Figure S2b,c, and Movies S11 and S12 of the Supporting Information), respectively.

**Figure 2:**
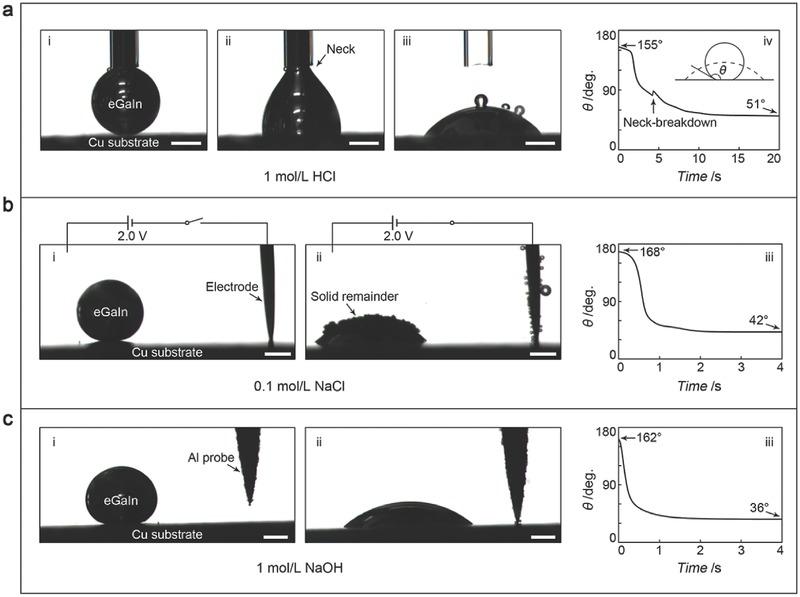
Wetting behavior and time‐dependent contact angle evolution of the LM droplets under different excitations: a) acidic solution with no additional assistance; b) neutral solution assisted by an electrical polarization; c) alkaline solution assisted by an aluminum probe. The subfigure on the right side of each row shows the time‐dependent contact angle evolution. The inset of figure a‐iv schematically shows the measured contact angle θ during the nonwetting‐to‐wetting transition. Scale bars: 500 µm.

We recognized that the nonwetting‐to‐wetting transitions and their timescales shown in Figure 2 were highly consistent with those of the LM‐Phagocytosis processes shown in Figure 1. Moreover, solid remainder was found gathering on the meniscus top in the NaCl solution but was absent in the HCl solution and the NaOH solution during contact angle measurements, which also matched well with the phenomena observed in LM‐Phagocytosis. All these consistencies showed unambiguously that the LM‐Phagocytosis was caused by the resulted wetting. However, one might notice that while the final wetting state of the LM droplets on the substrates exhibited a partial‐wetting configuration, the LM showed a complete‐wetting behavior to the metal particles. This inconformity implied that there existed major differences behind the two competitive‐wetting configurations (particle internalization and droplet on‐plate spreading).

To bridge the particle internalization behavior and the droplet on‐plate spreading process, we have further examined two theoretical cases that one is a spherical solid particle migrating from one liquid to another (case I) and the other is a liquid droplet spreading on a solid substrate (case II) in the same liquid environment (**Figure** **3**a–d). Following the geometrical constrains of each case, the total surface free energy of each system can be deduced and normalized as (see Supporting Information for derivation details)^24^, ^25^

(1) $$F_{I}\;\left(X\right)\;=\;X^{2}\;-\;\left(1\;-\;Y\right)X$$

(2) $$F_{\mathrm{II}}\left(X\right)\;=\;\frac{2\;+\;Y}{3}X^{2}\;+\;\frac{1\;-\;Y}{3}\;\frac{1}{X}$$

where *F*_I_(*X*) and *F*_II_(*X*) represent the surface free energy as a function of *X* for case I and case II, respectively. *X* = *h*/2*R*_0_, *Y* = λ(γ_2_ − γ_1_)/γ_12_, *h* is the height of the spherical cap for both cases, *R*_0_ is the radius of the particle for case I while *R*_0_ is the radius of liquid 1 before spreading (*h =* 2*R*_0_) for case II, λ is the Wenzel coefficient which characterizes the influence of surface roughness.^26^ By definition it imposes λ > 1 but not necessarily should λ be identical for the two cases. γ is the interfacial tension with subscripts indicating different interfaces (Figure 3a,c). The variable *X* and the apparent contact angle θ_W_ are equivalent measures of the wetting process for both cases and the wetting begins at *X* = 1 (θ_W_ = 180°) and ends at *X* = 0 (θ_W_ = 0°). At equilibriums, γ_12_cosθ_W,eq_ + γ_1_ = γ_2_ and we get

(3) $$Y\;=\;\cos\theta_{W,\;\mathrm{eq}}\;=\;\lambda \cos\theta_{Y,\;\mathrm{eq}}$$

where θ_W,eq_ and θ_Y,eq_ are the apparent equilibrium contact angle (Wenzel's model) and intrinsic equilibrium contact angle (Young's model), respectively. It can be found that while *F*_I_(*X*) always follows a parabolic law (Figure 3b), *F*_II_(*X*) is largely deviated from parabolic evolution by the term 1/*X*, except for θ_W,eq_ = 0° when *F*_I_(*X*) and *F*_II_(*X*) become identical (Figure 3d). Therefore, for non‐complete wetting conditions, while the surface energy needed to internalize a particle is a slow‐growing finite value, to further spread a sessile droplet on a plate will need a substantial amount of energy to overcome the fast‐growing surface free energy *F*_II_(*X*) as a result of the dominance of 1/*X* in contribution. Such physical interpretation decodes the inconformity in wetting configurations observed in our experiments.

**Figure 3:**
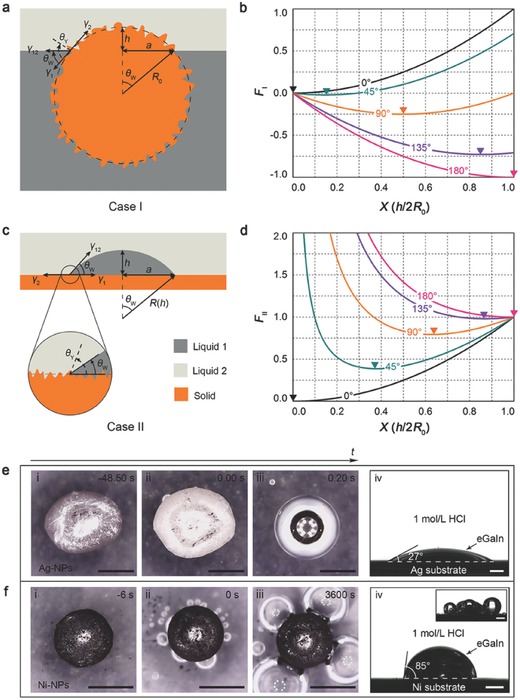
a) Schematic drawings of the particle internalization process and b) the corresponding surface free energy evolution; c) schematic drawings of the droplet on‐plate spreading process and d) the corresponding surface free energy evolution. The influence of surface roughness is enlarged for clarity and the minimums of *F*_I_(*X*) and *F*_II_(*X*) for each θ_W,eq_ are indicated by triangle markers. e) Comparative study with Ag‐NPs and f) Ni‐NPs. Note that figure f‐iv is photographed when the bubbles are gently removed since bubble generation becomes more intense when nickel substrate is used (see the inset figure). Scale bars: 500 µm.

The parabolic equation, Equation (1), has a symmetry axis at *X* = (1 − *Y*)/2 = (1 − cosθ_W,eq_)/2, where *F*_I_(*X*) reaches its minimum and θ_W_ = θ_W,eq_. Based on the energy minimum principle, particles will autonomously fit themselves to obtain this energetically favorable configuration in order to minimize the total energy of the system. Therefore, the energy barrier for particle internalization should be the energy gradient between the complete‐wetting state and the equilibrium state featured by θ_W,eq_. Our simple model also provides a straightforward derivation of this energy barrier from Equation (1)

(4) $$\Delta E\;=\;4\pi R_{0}^{2}\gamma_{12}{F_{I}(x)|}_{\mathrm{eq}}^{0}\;=\;\pi R_{0}^{2}\gamma_{12}{\left(1\;-\;\cos\theta_{W,\mathrm{eq}}\right)}^{2}$$

According to Equation (4), the energy barrier Δ*E* can be calculated if information about the particle geometry, LM‐solution interfacial tension and the equilibrium contact angle is provided. For spherical particles of 10 um and 10 nm in diameter, taking γ_12_ to be 0.6 Nm^−1^ and θ_W,eq_ to be 45°, the energy barrier is estimated to be on the scale of 10^−12^ J and 10^−18^ J, respectively.

As for the whole nonwetting‐to‐wetting transition for a given θ_W,eq_, *F*_I_(*X*) decreases along with the decrease of *X* at the right side of its symmetric axis where θ_W_ > θ_W,eq_, and *F*_I_(*X*) increases as *X* further decreases along the left side of its symmetric axis where θ_W_ < θ_W,eq_. In ideal conditions, if θ_W,eq_ < 90°, $F_{I}(X){|}_{1}^{0}\;<\;0$, the surface free energy generated during the nonwetting‐to‐wetting transition exceeds Δ*E*, then autonomous particle internalization can be achieved. A more intuitive analogy to such situation is a free‐falling roller‐coaster which rushes through its rail bottom and then climbs on to an intermediate level. On the contrary, if θ_W,eq_ > 90°, $F_{I}(X){|}_{1}^{0}\;>\;0$, which results in insufficient energy for particle internalization and therefore, additional energy should be provided to assist the process. Only when θ_W,eq_ = 90°, the two cancel each other out. This implies that LM‐Phagocytosis not just requires a wettability alteration, but also depends on to what extent the equilibrium wetting state can be achieved.

Our theoretical model was valid for copper on which contact angles of no more than 51° were measured. For further validation, comparative studies using silver and nickel NPs were conducted in HCl solution. The contact angle of the LM on the silver substrate was identified to be 27° (Figure 3e‐iv), which was much smaller than that on the copper substrate. Based on our theory, due to a smaller energy barrier and a more significant surface free energy decrease, a more abrupt particle internalization process (on the timescale of a few tenths of a second) was in accordance with expectation (Figure 3e, and Movie S13 of the Supporting Material). For the nickel NPs, particle internalization process did not take place even after as long as 1 h (Figure 3f, and Movie S14 of the Supporting Information). After contact angle measurement, the contact angle of the LM on the nickel substrate was measured to be 85°, which was very close to 90°. Given that in real cases, the nonwetting‐to‐wetting transition began at a contact angle smaller than 180°, the equilibrium contact angle should be sufficiently smaller than 90° to induce particle internalization. So it was also reasonable to find out that the nickel particles were not internalized.

It was disclosed that the surface free energy generated from the wettability alteration was capable of internalizing the solid metal particles if adequate wetting could be achieved. However, certain “force” must be presented to sustain the state because being internalized was not the most energetically favorable configuration, which would be obtained when the contact angle reached θ_W,eq_ (Young's law). We found that this ‘force' stemmed from the inherent reactive nature of intermetallic wetting. Different from reversible nonmetallic wetting conditions,^27^ intermetallic wetting was usually irreversible and accompanied with intermetallic‐phase formation.^28^, ^29^ Formation of intermetallic phase (metallic bond) was a much stronger effect than intermolecular interactions,^24^ and therefore, the reactive nature of the wetting process should be effective to keep the particles being internalized. Evidence for intermetallic wetting between the LM and the copper substrate could be found in Figure S3a–d of the Supporting Information.

It was also established that metal surfaces, liquid or solid, would gradually develop a passivated oxide layer in air.^30^ So in order to achieve reactive wetting, it was necessary to break down the impeditive oxide layers on the surface of both the LM and the particles. At this point, when we recalled the observed phenomena related to color and shape transitions all together with the black remainder in the experiments, the differences caused by the pH of the solutions could be well explained. The oxides of the LM (Ga_2_O_3_ and In_2_O_3_) were amphoteric oxides but copper oxides (Cu_2_O and CuO) were alkaline products. Both amphoteric and alkaline oxides could be dissolved in an acidic medium but only the former could be dissolved in an alkaline medium. Consequently, the HCl solution could remove both oxide layers and trigger LM‐Phagocytosis without additional assistance. The color transition was a result of the removal of oxides on the particles. And the removal of LM oxides, which recovered its surface tension, was responsible for the observed shape transition. Moreover, it should be the electrical polarization that broke down both oxide layers in the NaCl solution and the remained particle oxide layer in the NaOH solution, respectively, in order to achieve intermetallic contact. Cathode reduction^31^ and electrical breakdown of dielectric films^32^ could be the mechanisms for the electrical polarization to take effect. These analyses also suggested that the black remainder found in the NaCl solution was mainly LM oxides since they were nonreactive with neutral medium and couldn't be recovered via cathode reduction, neither. The evident change in color and surface morphology of the copper substrates provided further proof for the surface transitions of the particles (Figure S3e–j of the Supporting Information).

In summary, we demonstrated a particle internalization effect in gallium‐based LMs together with three kinds of methods to realize it. The method of using an electrical polarization revealed as a powerful and easily adjustable way. Therefore, it could be applied as long as the solution was electrically conductive. A theoretical model was built to assist interpreting the mechanisms and it showed good consistency with experiments. The equilibrium contact angle and the reactive nature of intermetallic wetting were believed to be two key factors for the particle internalization process. The fact that these two factors were both intrinsically determined by material nature explained why LM‐Phagocytosis was material‐selective. This study revealed a fundamental interfacial‐to‐internal intermetallic process across the LM‐solution boundary. With the knowledge it provided here, the energy barrier of LM‐Phagocytosis for different materials can be estimated and various LM‐particle mixtures can also be prepared by using the proposed methods, either separately or in a combined manner.

## Experimental Section

The liquid metal was prepared by stirring and heating gallium (75.5 wt%, 99.99% purity) and indium (24.5 wt%, 99.99% purity) together to 80 °C for 2 h until eutectic metal alloy was formed. The concentrations of the HCl solution, NaCl solution, and NaOH solution used throughout the experiments were 1, 0.1, and 1 mol L^−1^, respectively. Characterizations of the metal particles that were used in the experiments were presented in Figure S4 (Supporting Information). All the experiments were conducted at room temperature.

## Conflict of Interest

The authors declare no conflict of interest.

## Supporting information

Supplementary

Supplementary

Supplementary

Supplementary

Supplementary

Supplementary

Supplementary

Supplementary

Supplementary

Supplementary

Supplementary

Supplementary

Supplementary

Supplementary

Supplementary
