# Supplementary material for: Liquid Metal Phagocytosis: Intermetallic Wetting Induced Particle Internalization
Source: Adv Sci (Weinh). 2017 Apr 24;4(5):1700024. doi: 10.1002/advs.201700024 (PMC5441499; doi:10.1002/advs.201700024)
Supplement: Supplementary file 1 — Supplementary [file ADVS-4-na-s001.pdf]

Copyright WILEY-VCH Verlag GmbH & Co. KGaA, 69469 Weinheim, Germany, 2017.

ADVANCED SCIENCE
Open Access

Supporting Information

for *Adv. Sci.,* DOI: 10.1002/advs.201700024

Liquid Metal Phagocytosis: Intermetallic Wetting Induced Particle Internalization

*Jianbo Tang, Xi Zhao, Jing Li, Yuan Zhou, and Jing Liu**

Advanced Science

Supporting Information

Liquid Metal Phagocytosis: Intermetallic Wetting Induced Particle Internalization

Dr. J. Tang, Prof. J. Liu* (E-mail: jliubme@tsinghua.edu.cn)
Department of Biomedical Engineering, School of Medicine,
Tsinghua University, Beijing 100084, China

Ms. X. Zhao, Prof. Y. Zhou, Prof. J. Liu
Key Laboratory of Cryogenics, Technical Institute of Physics and Chemistry,
Chinese Academy of Sciences, Beijing 100190, China

Dr. J. Li
Key Laboratory of Photochemical Conversion and Optoelectronic Materials, Technical Institute of Physics and Chemistry,
Chinese Academy of Sciences, Beijing 100190, China

Figure S1. Liquid metal marbles coated with Cu-MPs and Cu-NPs in neutral and alkaline solution without additional assistance: a) NaCl solution; b) NaOH solution. The subfigures i-iii in each row are time-lapse images extracted from the movies with zero time indicating the beginning of adding solution. Scale bars: 200 µm.

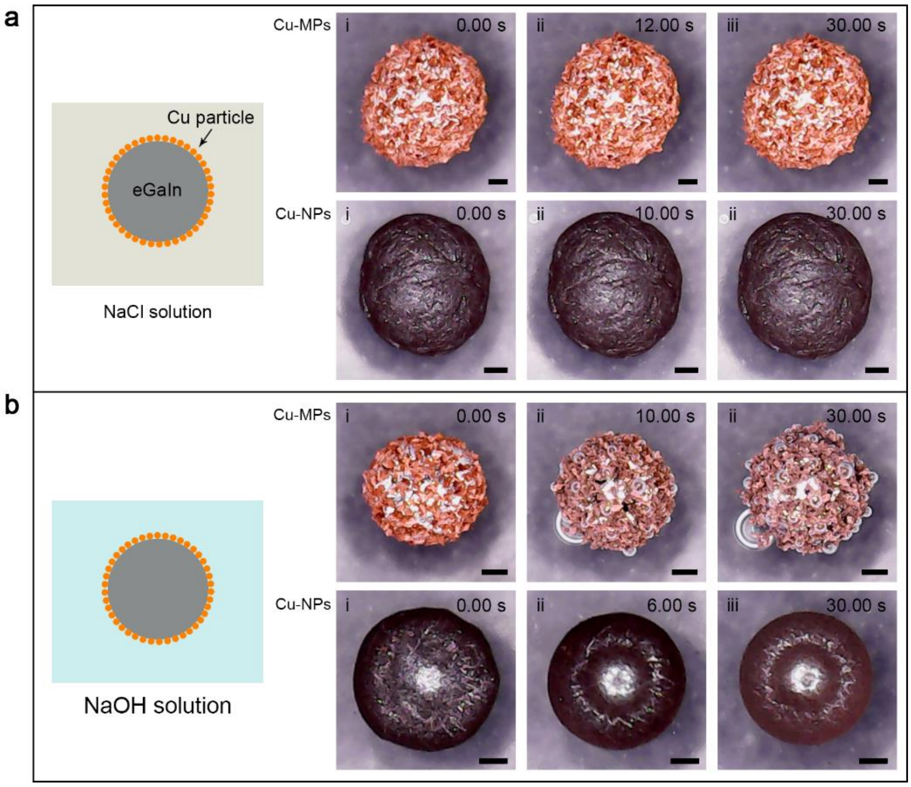

Figure S2. a) Liquid metal phagocytosis realized in NaOH solution assisted by an electrical polarization. The subfigures i-iv in each row are time-lapse images extracted from the movies with zero time indicating the beginning of electrical polarizing; b) Wetting behavior and time-dependent contact angle evolution of the liquid metal on the copper substrate in the NaOH solution assisted by an electrical polarization. Scale bars: 500 μm.

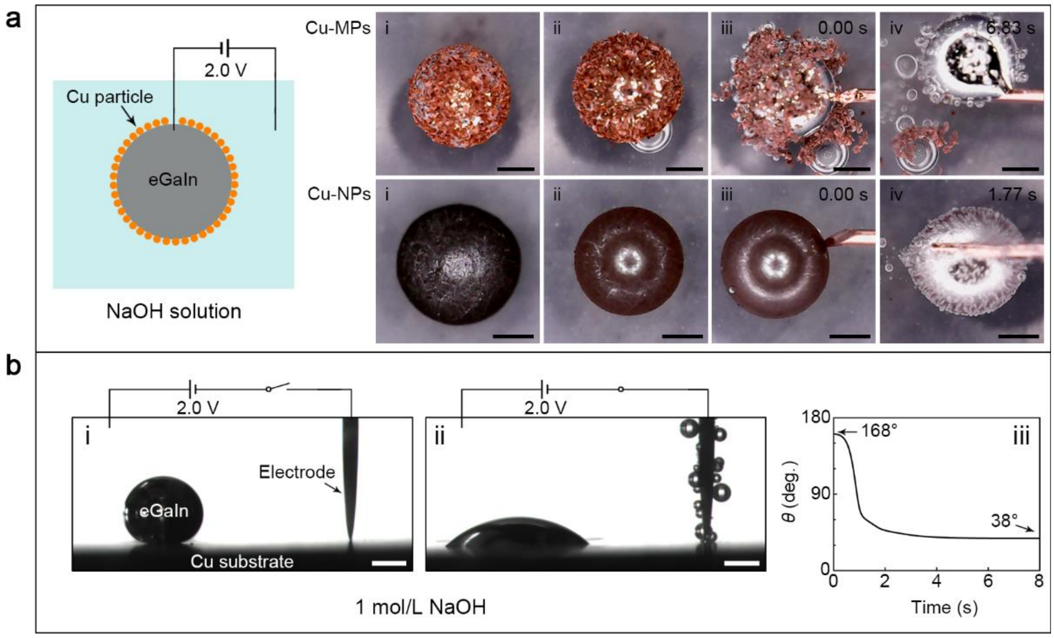

Figure S3. a)-d) Characterization of intermetallic wetting: a) A photograph shows the circular liquid-metal-wetted region after the liquid metal being wiped; b) SEM characterization of reactive wetting featuring the wetting boundary region and the corresponding: c) Mapping of Ga; d) Mapping of Cu. Distinct distribution differences exist between Ga and Cu divided by the contact line. The coexistence of the solid metal and the liquid metal compositions in the wetted region was an indication of reactive wetting. e)-j) Characterization of surface transition: Schematic drawing and photographs of the copper substrate partially treated by: e) HCl solution with no additional assistance; f) NaCl solution assisted by an electrical polarization, and g) NaOH solution assisted by a sacrificial aluminum probe; h) Typical SEM characterization of surface transition of the copper substrate; i) Magnified untreated region; j) Magnified treated region.

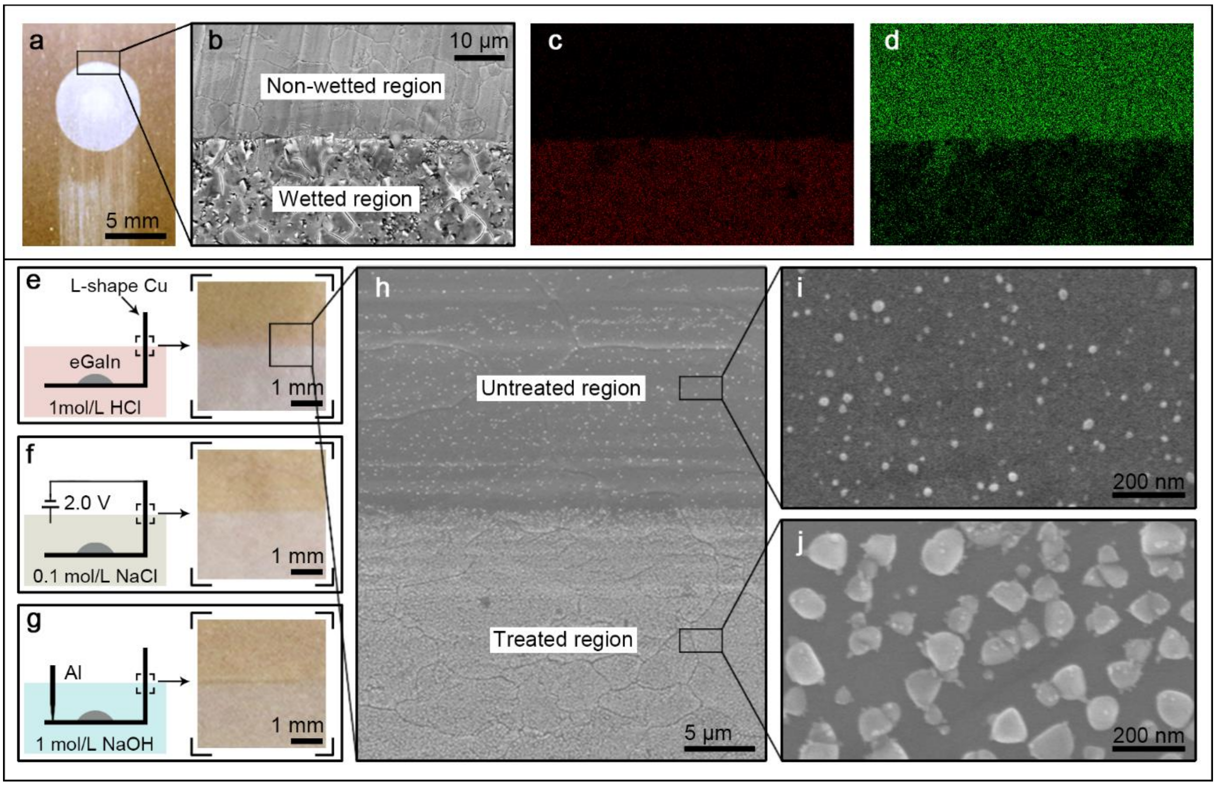

Figure S4. Characterization of metal particles: a) Micro copper particles; b) Nano copper particles; c) Nano silver particles. Note that the nickel particles are not characterized due to the difficulties brought by the magnetism of the material to the SEM equipment. All the particles are purchased from Beijing DK Nano Technology Co., Ltd., according to which the average size of the nano nickel particles is 50 nm.

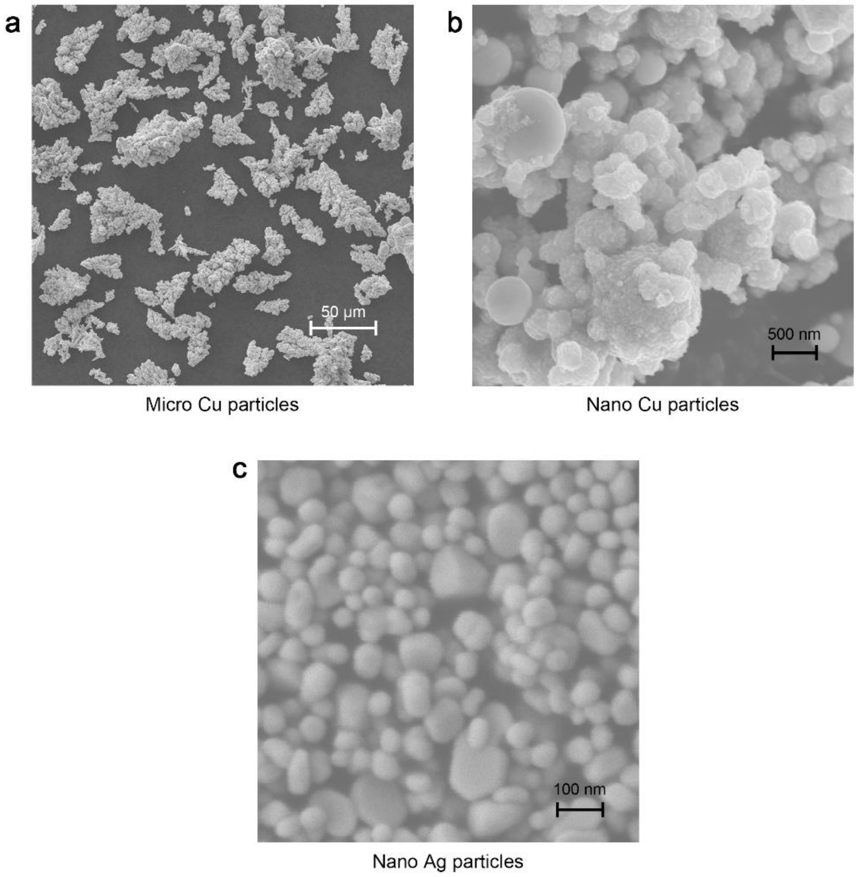

Derivation of Equation (1) and (2)

For the two cases of a spherical solid particle migrating from one liquid phase to another (case I, **Figure 3**a) and a liquid droplet spreading on a solid substrate (case II, Figure 3c) in the same liquid, the total surface free energy of both systems can be interpreted as the sum of the three surface free energy terms: [1, 2]

$$U_{\mathrm{surf}} = \overline{A}_{12}\gamma_{12} + \lambda\overline{A}_{\mathrm{I}}\gamma_1 + \lambda\overline{A}_2\gamma_2 \quad (S1)$$

where $\overline{A}$ is the apparent contact area, λ is a coefficient that characterizes the solid-surface roughness. By definition it imposes $\lambda > 1$ but not necessarily should λ be identical for the two cases. γ is the interfacial tension. The subscripts 12, 1 and 2 denoting the liquid 1-liquid 2 interface, the liquid 1-solid interface, and the liquid 2-solid interface, respectively. By doing so, assumptions are made that the contribution of gravity (and buoyancy) is insignificant and the two liquids are both Newtonian fluids, under which elastic energy is absent.[2, 3] When the height of the spherical cap of the two cases is h, the surface area of the curved spherical caps and their bases are $2\pi Rh$ and πa^2, respectively. Then the total surface free energy U_{surf} of case I and case II can be written as follows:

$$U_{\mathrm{surf,I}} = (A_{12} - \pi a^2)\gamma_{12} + 2\pi R(2R - h)\lambda\gamma_1 + 2\pi Rh\lambda\gamma_2 \quad (S2a)$$

$$U_{\mathrm{surf,II}} = 2\pi Rh\gamma_{12} + \pi a^2\lambda\gamma_1 + (A_2 - \pi a^2)\lambda\gamma_2 \quad (S2b)$$

where A_{12} denotes the contact area of the liquid 1-liquid 2 interface of case I when $h = 2R_0$ and A_2 denotes the liquid 2-solid interface of case II also when $h = 2R_0$, respectively. For case I, $R = R_0$ is the radius of the particle which remains a constant as h varies. But for case II, R is the radius of the spherical cap which is a function of h and R_0 is the radius of the liquid metal droplet before contacting ($h = 2R_0$). And for both cases, a is the radius of the base of the spherical caps. By introducing the geometrical constrains $R^2 = (R - h)^2 + a^2$ for

both cases and $4\pi R_0^3/3 = \pi h(3a^2 + h^2)/6$ (incompressibility) for case II, Equations (S2a) and (S2b) then become:

$$U_{\mathrm{surf,I}}(h) = \pi\gamma_{12}h^2 - 2\pi R_0(\gamma_{12} + \lambda\gamma_1 - \lambda\gamma_2)h + 4\pi {R_0}^2\lambda\gamma_1 + A_{12}\gamma_{12} \qquad \text{(S3a)}$$

$$U_{\mathrm{surf,II}}(h) = \frac{\pi(2\gamma_{12} - \lambda\gamma_1 + \lambda\gamma_2)}{3}h^2 + \frac{8\pi R_0^3(\gamma_{12} + \lambda\gamma_1 - \lambda\gamma_2)}{3}\frac{1}{h} + A_2\lambda\gamma_2 \qquad \text{(S3b)}$$

Then we introduce the dimensionless quantities as follows:

$$X = \frac{h}{2R_0}, X \in [0,1] \qquad \text{(S4a)}$$

$$Y = \frac{\gamma_2 - \gamma_1}{\gamma_{12}} \qquad \text{(S4b)}$$

$$Z = \frac{\gamma_2 + \gamma_1}{\gamma_{12}} \qquad \text{(S4c)}$$

$$C_{\mathrm{I}} = \lambda\frac{Z - Y}{2} + \frac{A_{12}}{4\pi R_0^2} \qquad \text{(S4d)}$$

$$C_{\mathrm{II}} = \lambda\frac{A_2}{4\pi R_0^2}\frac{Y + Z}{2} \qquad \text{(S4e)}$$

$$\widetilde{U}_{\mathrm{surf}} = \frac{U_{\mathrm{surf}}}{4\pi R_0^2\gamma_{12}} \qquad \text{(S4f)}$$

$$F = \widetilde{U}_{\mathrm{surf}} - C \qquad \text{(S4g)}$$

Further dividing Equation (S3a) and (S3b) by $4\pi R_0^2\gamma_{12}$ produces:

$$F_{\mathrm{I}}(X) = \widetilde{U}_{\mathrm{surf,I}} - C_{\mathrm{I}} = X^2 - (1 - \lambda Y)X \qquad \text{(S5a)}$$

$$F_{\mathrm{II}}(X) = \widetilde{U}_{\mathrm{surf,II}} - C_{\mathrm{II}} = \frac{2 + \lambda Y}{3}X^2 + \frac{1 - \lambda Y}{3}\frac{1}{X} \qquad \text{(S5b)}$$

Since $Y = (\gamma_2 - \gamma_1)/\gamma_{12} = \cos\theta_{\mathrm{Y,eq}}$, the influence of surface roughness can be also expressed as the change of the intrinsic equilibrium contact angle $\cos\theta_{\mathrm{Y,eq}}$ (Young's model) to the apparent equilibrium contact angle $\cos\theta_{\mathrm{W,eq}}$ (Wenzel's model), where $\cos\theta_{\mathrm{W,eq}} = \lambda\cos\theta_{\mathrm{Y,eq}}$. Considering that during contact angle measurements, it is $\cos\theta_{\mathrm{W,eq}}$, not $\cos\theta_{\mathrm{Y,eq}}$ that is measured, which means the influence of surface roughness is implicitly included, we use $Y' = \lambda Y$ to replace λY and $Z' = \lambda Z$ to replace λZ, respectively. And finally we get:

$$F_{\mathrm{I}}(X) = X^2 - (1 - Y')X \qquad \text{(S6a)}$$

$$F_{\mathrm{II}}(X) = \frac{2+Y'}{3}X^2 + \frac{1-Y'}{3}\frac{1}{X} \tag{S6b}$$

in which case $C'_{\mathrm{I}} = \frac{Z'-Y'}{2} + \frac{A_{12}}{4\pi R_0^2}$ and $C'_{\mathrm{II}} = \frac{A_2}{4\pi R_0^2}\frac{Y'+Z'}{2}$.

Reference

[1] J. N. Israelachvili, *Intermolecular and Surface Forces*, 3rd edition, Academic Press, Amsterdam, The Netherlands **2011**, p. 415.

[2] T. Salez, M. Benzaquen, E. Raphael, *Soft Matter* **2013**, *9*, 10699.

[3] Z. Cao, M. J. Stevens, A. V. Dobrynin, *Macromolecules* **2014**, 47, 3203.
